# Enhancing rural veterinary governance: coupling veterinary human resources with public attention to animal welfare

**DOI:** 10.3389/fvets.2025.1661775

**Published:** 2025-09-25

**Authors:** Xianhang Xu, Hong Liu, Jiejing Yang, Mohd Anuar Arshad, Yinglei He, Qianqian Chen, Chenshu Yu

**Affiliations:** ^1^School of Management, Chongqing Institute of Engineering, Chongqing, China; ^2^School of Management, Universiti Sains Malaysia, Penang, Malaysia; ^3^School of Economics and Management, Tianjin Tianshi College, Tianjin, China; ^4^School of Business, Henan Kaifeng College of Science Technology and Communication, Kaifeng, China; ^5^School of Foreign Languages and Literature, Heilongjiang University, Harbin, China

**Keywords:** veterinary human resources, animal welfare, public awareness, coupling coordination degree, rural governance

## Abstract

**Objective:**

This study aims to evaluate the coordination between veterinary human resources (VHR) and public awareness of animal welfare (PAAW) within the context of rural veterinary governance in China. It investigates how mismatches between institutional service capacity and public demand affect the development of animal health systems and human–animal relationships.

**Methods:**

The study proposes a framework to examine the coupling between veterinary service supply and public awareness through the VHR and PAAW subsystems across 31 Chinese provinces from 2014 to 2023. It calculates annual scores using the entropy weight method, checks causal links with a panel vector autoregression (PVAR) model, and assesses coordination using the coupling coordination degree (CCD) model to assess their alignment. The relative development degree (RDD) model is further applied to evaluate development imbalances, while geographic information system (GIS) mapping visualizes spatial coordination patterns.

**Results:**

PAAW has steadily increased in most areas, while VHR has grown more slowly. There is a unidirectional relationship where VHR significantly drives PAAW, but the reverse effect is weak. The overall CCD remains low, with most provinces showing a “VHR Lag” type, pointing to structural imbalances in supply–demand coordination.

**Conclusion:**

The study highlights the need to enhance the veterinary workforce and promote public engagement to improve animal health governance. The proposed framework offers a transferable tool for assessing implementation gaps and regional disparities in veterinary service provision, contributing to integrated approaches that connect veterinary systems, public awareness, and rural development.

## 1 Introduction

Public interest in human–animal relationships has grown noticeably in recent years (1). The quality of relationships affects not only animals’ survival but also human emotional support, mental health, and social ethics (2). Ensuring both physical health and emotional well-being of animals has become increasingly important, and animal welfare is now studied across many different fields (3). Most research so far has looked at things like animal nutrition, disease control, and living conditions. But there has been little focus on how public awareness, service systems, and animal welfare work together as a whole (4, 5). One major problem is public needs and service supply often do not match, which has become a serious barrier to putting animal welfare into practice (6).

In rural and developing areas, local veterinary services are the main support for animal care (7). They are responsible for preventing disease outbreaks, treating common diseases, and guiding animal husbandry practices (8). How well they work mainly depends on human resources—how many veterinarians are available, how skilled they are, and how they are rewarded (9). At the same time, more people are paying attention to animal health and welfare. This is evident in the frequency of online search activities and digital discourse (10). These actions are now a key driving force for improving public services and animal care (11).

In this context, rural veterinary governance has become a critical link in advancing animal health and public interest (12). There is growing recognition that public attention and veterinary service capacity must be jointly considered to ensure responsive and inclusive animal health systems (13). Many countries are now bringing veterinary services, disease control, and public awareness into one system to build stronger and more responsive health systems (14). Under this approach, public attention to animal welfare (PAAW) is not just about ethics. It also affects public safety and the ability of systems to respond (15).

However, most current studies still examine veterinary human resources (VHR) and PAAW as separate dimensions. They often miss the possible link between supply and demand, and the risks of mismatch across regions. In rural areas, especially, the lack of information and limited resources make this imbalance worse. These gaps hinder effective coordination between veterinary infrastructure and public expectations at the local level.

To address this issue, this study removes the emphasis on theoretical framings, such as One Health, and instead focuses on the practical coordination between service provision and public concern in rural animal health governance. It develops an analysis framework by combining statistical yearbook data with online search data to examine the coupling dynamics between service supply and public awareness. Focusing on the VHR and PAAW subsystems, the study examines their development, relationship, and coordination across regions. While centered on the case of China, the study also aims to provide insights for developing countries seeking to build data-driven, public-oriented animal health systems. Accordingly, the study raises the following research questions (RQs):

*RQ1*: How can a multi-dimensional index system be constructed and applied to assess the levels of VHR and PAAW?

*RQ2*: Is there a causal relationship between VHR and PAAW?

*RQ3*: What are the spatiotemporal characteristics of coordination between VHR and PAAW?

This study develops a framework linking veterinary services and public awareness through the VHR and PAAW subsystems. In this way, it moves animal welfare research from focusing on just one side to looking at how the whole system works together. On the practical side, it evaluates how well local veterinary services and public concern are aligned across regions, offering evidence to support better resource allocation and targeted governance strategies. In terms of methods, the study combines the entropy weight method, panel vector autoregression (PVAR), the coupling coordination degree (CCD) model, and geographic information system (GIS) mapping. This shows the potential for cross-disciplinary work that links animal science, rural governance, and public data analysis, offering insights into how veterinary services can better respond to social needs in underserved regions.

## 2 Literature review

### 2.1 Veterinary human resources

VHR is a key part of the animal health system on the supply side (16). It includes the quantity, quality, structure, and allocation efficiency of veterinary professionals (17). VHR directly affects the level of technical support and public services in livestock development and plays an important role in promoting sustainable agriculture (18). As a vital link between policy and local practice, veterinary stations take on basic tasks in disease prevention, animal health, and rural public services (19).

Current research mainly focuses on the quantity and structure of VHR. Common indicators include the number of veterinary personnel per thousand livestock, the education level of personnel, and the share of senior professional titles (20). However, the real challenges go beyond staff shortages. They also include the aging of rural veterinary teams, low job appeal, and limited promotion opportunities (21). These issues have led to weaker professional capacity, lower motivation to serve, and higher turnover among veterinary personnel (22).

To better reflect the structure and capacity of veterinary teams, recent studies have started to include factors like performance output and incentive systems (17). For example, the work efficiency of each staff member can show how well human resources are used and how skills are applied in practice (23). Income levels reflect job attractiveness and policy support, both of which matter for staff stability and long-term service delivery (24). The way performance and incentives work together has become an important factor affecting the quality of local veterinary services (25).

Overall, most studies on VHR focus on static analysis of quantity and structure. They rarely consider the combined effects of factors like performance and incentives, and few offer a systematic evaluation framework. As a result, it is hard to fully capture the differences and real issues in human resource allocation across regions. Therefore, it is necessary to build a comprehensive index with multiple indicators that reflect regional features and changing trends. This can help improve the quality of public veterinary services and support better talent planning.

### 2.2 Public attention to animal welfare

PAAW shows how people think and feel about animal health, medical care, and related services (26). It is increasingly seen as an important measure of social progress and public governance capacity (27). With the rapid growth of digital technology, studies on public attention no longer rely only on surveys or interviews. They also use online behavior data and tools, such as search trends, social media content, and digital interactions (28).

Studies have found that factors such as media coverage, unexpected events, and government responses often have a strong impact on PAAW issues (29). This is especially clear in cases related to animal disease outbreaks, food safety incidents, or animal abuse (30). For example, events like African swine fever or problems with livestock products often lead to a sharp rise in search activity, showing the emotional sensitivity and public resonance of animal welfare topics (31, 32).

On the methodological side, platforms like Google Trends, Twitter, and Baidu Index are widely used to track the time patterns and geographic spread of public attention. These tools help reveal regular cycles and regional differences in interest (33). In areas with higher internet use and more advanced urbanization, public attention tends to be more consistent, rational, and structured (34).

Although existing research has made progress in both theory and method, most work still focuses on responses to specific events or general trend analysis (35). There is limited attention to how public awareness connects with local public services. Recently, some scholars have begun to study how people understand animal diseases and respond to prevention services, trying to learn more about their needs and habits (36). This provides new ideas for building an index that can measure public awareness while reflecting regional differences. It also helps explore how public attention relates to the allocation of veterinary service resources.

### 2.3 The relationship between VHR and PAAW

VHR and PAAW form a supply–demand relationship in animal health governance (37). On the supply side, a strong and skilled veterinary team is key for disease control, welfare checks, and emergency services (15, 38). This is especially important in rural areas, where the quantity, ability, and motivation of local veterinarians directly affect service access and response speed (14). On the demand side, public awareness for animal health shows up not only in emotions and online searches, but also in public opinion and social pressure that can push for better policies and services (13, 39).

Recent studies have started to examine the relationship between VHR and PAAW, drawing on the One Health framework or online data sources (40, 41). However, much of this research remains fragmented and lacks systematic analysis, often treating VHR and PAAW as separate issues without exploring how they interact or whether supply and demand are aligned across regions. Therefore, it is necessary to build an integrated framework that connects VHR and PAAW. This can help identify supply–demand coordination across regions and support more precise and adaptive animal health governance.

## 3 Methodology

### 3.1 Research design

**Figure 1:**
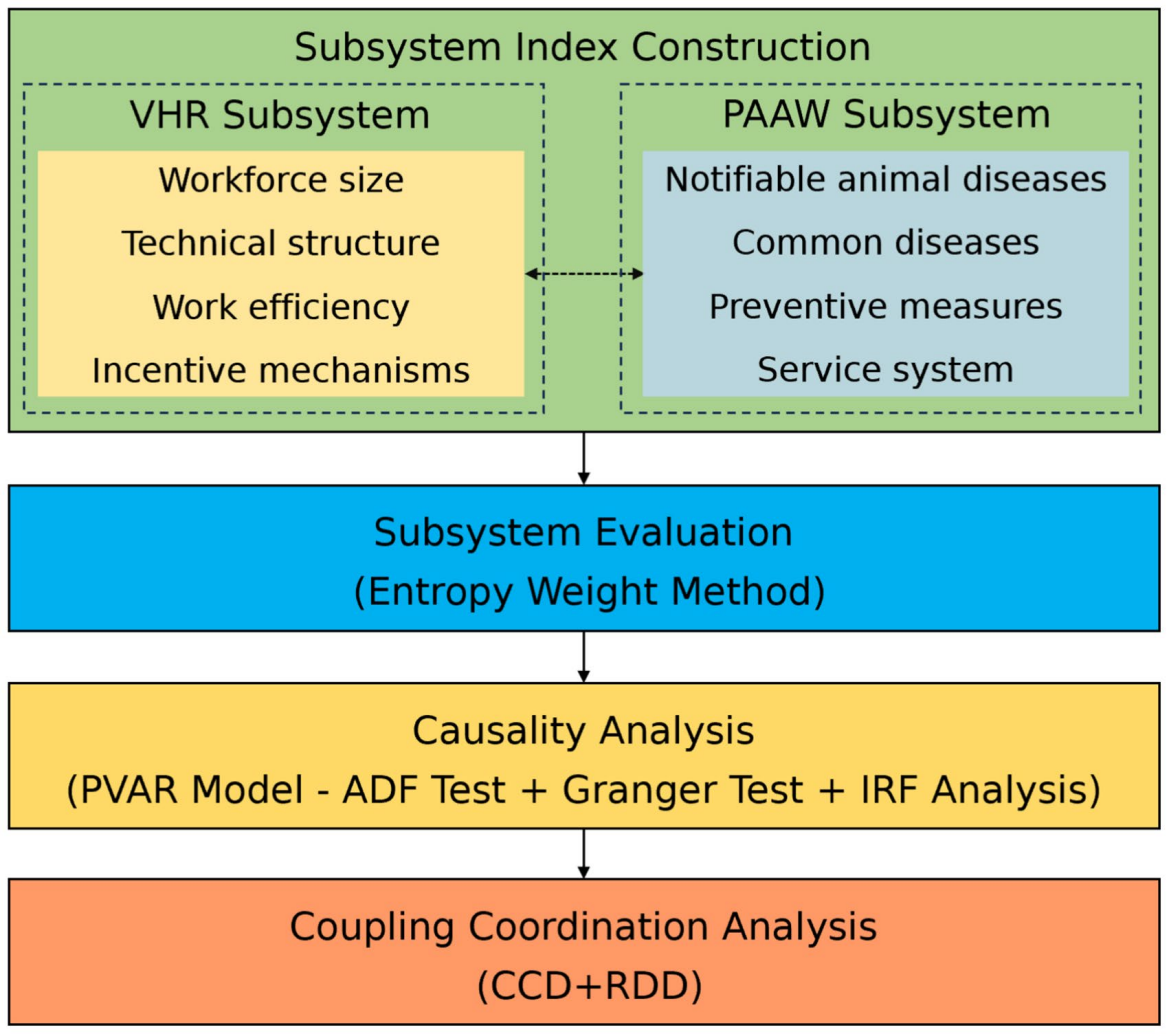
Research process.

The study aims to assess the development of VHR and PAAW across Chinese provinces from 2014 to 2023 and to uncover their causal relationship and coupling patterns. Based on the two dimensions of service supply and public awareness, a dual-system framework is developed. Using data from different sources and several quantitative methods, the study follows four main steps: constructing subsystem index, evaluating each subsystem, conducting causality analysis, and performing coupling coordination analysis (see Figure 1).

### 3.2 Research scope and data sources

**Figure 2:**
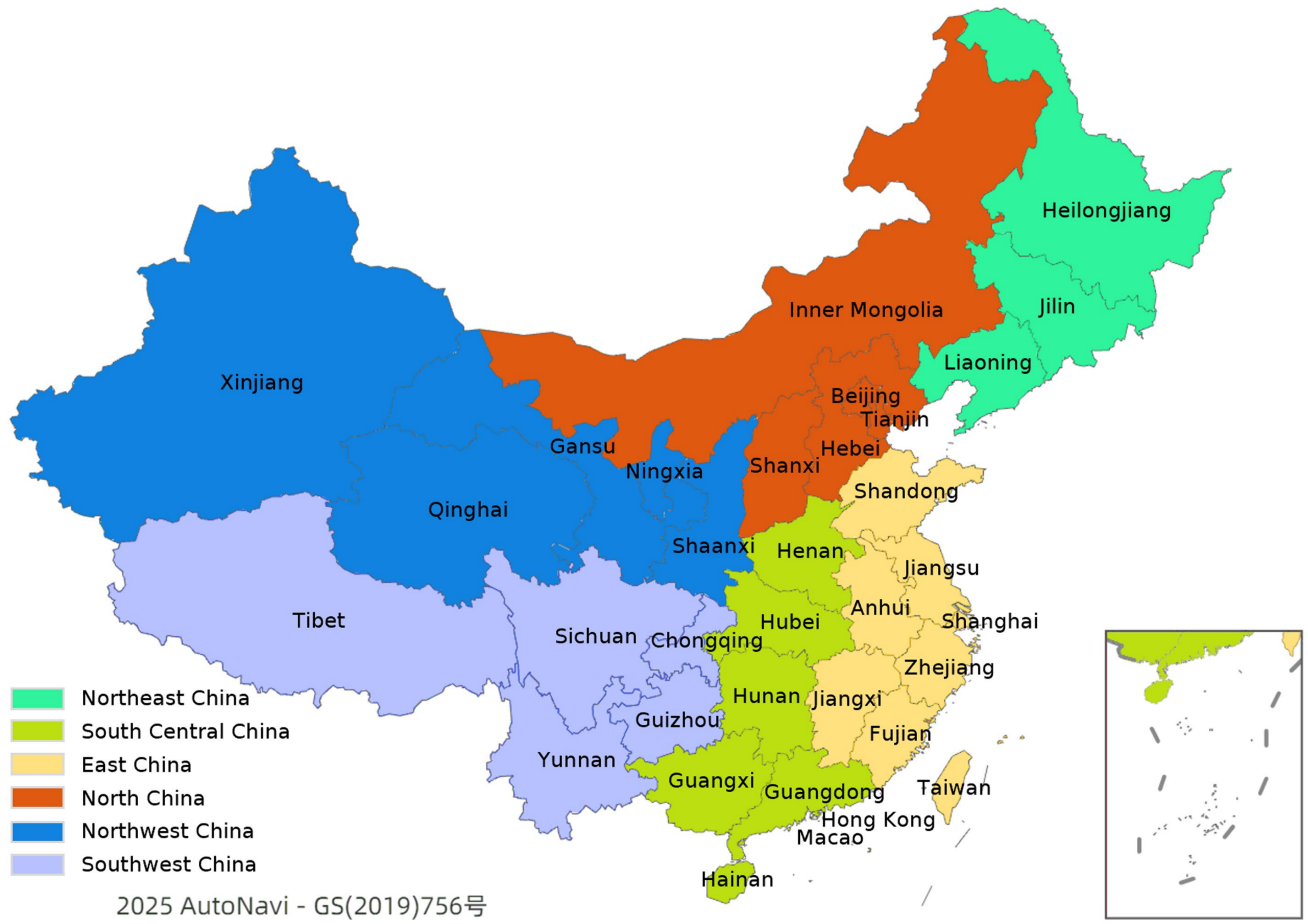
List of regions of China.

While this study includes all 31 provinces of mainland China, it also refers to the conventional six-region geographic division (42) (see Figure 2) for basic spatial orientation. However, all analyses are conducted at the provincial level, which better reflects policy implementation and veterinary governance structures.

Data on VHR are sourced from the *China Animal Husbandry and Veterinary Yearbook*, available in the CNKI Yearbooks Database.^1^ This includes information on the number of staff in township-level animal husbandry and veterinary stations (TAHVS) across provinces, as well as their technical structure, work efficiency, and salary levels. These indicators provide a comprehensive view of the supply characteristics of VHR in terms of quantity, quality, and efficiency. PAAW data come from Baidu Index.^2^ Public attention is measured by tracking search interest for eight keywords closely related to animal welfare: “avian influenza (禽流感),” “foot-and-mouth disease (口蹄疫),” “pig disease (猪病),” “chicken disease (鸡病),” “animal quarantine (动物检疫),” “animal epidemic prevention (动物防疫),” “animal husbandry and veterinary (畜牧兽医),” and “livestock station (畜牧站).” All data were retrieved in Mandarin at the provincial level using the Baidu Index platform and are collected annually from 2014 to 2023 for use in index construction and coupling analysis.

### 3.3 Evaluation index construction

#### 3.3.1 VHR subsystem

As noted earlier, VHR is a key factor in ensuring animal health and supporting the development of the livestock industry (18). Differences in the quantity, structure, skill, and incentives of VHR directly affect the accessibility and professionalism of veterinary services at the local level (17). Based on this, the study builds a province-level evaluation index of VHR to measure regional differences in human resource allocation and service capacity.

Considering both data availability and comparability across provinces, the VHR subsystem is designed around four dimensions: workforce size, technical structure, work efficiency, and incentive mechanisms (see Table 1). These indicators aim to capture how VHR supports livestock development and rural veterinary services across different regions.

**Table 1:** Evaluation index of VHR subsystem.

Dimension	Indicator	Unit	Attribute
Workforce size	Number of staff in TAHVS	Person	+
Technical structure	Proportion of staff with senior professional titles in TAHVS	%	+
Work efficiency	Annual average output per staff in TAHVS	RMB	+
Incentive mechanisms	Annual average salary of staff in TAHVS	RMB	+

Specifically, the workforce size dimension is measured by the number of staff in TAHVS, reflecting the overall supply capacity of local human resources in each region (43). The technical structure dimension is represented by the proportion of staff with senior professional titles, indicating the technical composition and qualification level of local veterinary teams (44). The work efficiency dimension is assessed by the annual average output per staff, which reflects the economic contribution per person and the service performance of the workforce (45). The incentive mechanisms dimension is measured by the annual average salary of staff, representing the strength of institutional support and the attractiveness of the position (46).

Unlike existing studies that focus mainly on static workforce size or educational background, this study incorporates efficiency and incentive dimensions to enable a more comprehensive assessment of VHR. The index covers four aspects: quantity, structure, efficiency, and incentives. It gives a clear picture of the overall strength of local veterinary teams in different regions and provides a solid base for the next-step coupling analysis with PAAW.

#### 3.3.2 PAAW subsystem

As discussed above, PAAW has become an important social force driving improvements in local veterinary service systems (26). Public attention to animal diseases, prevention behaviors, and institutional services not only reflects responsiveness at the level of information behavior but also reveals potential preferences for services and a willingness to participate in governance (27). Therefore, building an index that can quantify and describe public attention across multiple dimensions helps identify how well supply and demand are aligned and where structural mismatches may exist.

This study draws on the three-dimensional animal welfare framework proposed by the World Organisation for Animal Health (WOAH), which includes physical health, emotional and mental state, and expression of natural behaviors (47). Based on this, the PAAW subsystem is developed using four dimensions: notifiable animal diseases, common diseases, preventive measures, and the service system. The subsystem is designed to reflect both the rural context and the characteristics of online behavior (see Table 2).

**Table 2:** Evaluation index of PAAW subsystem.

Dimension	Indicator	Unit	Attribute
Notifiable animal diseases	Avian influenza	Baidu Index (points)	+
	Foot-and-mouth disease	Baidu Index (points)	+
Common diseases	Pig diseases	Baidu Index (points)	+
	Chicken diseases	Baidu Index (points)	+
Preventive measures	Animal quarantine	Baidu Index (points)	+
	Animal epidemic prevention	Baidu Index (points)	+
Service system	Animal husbandry and veterinary	Baidu Index (points)	+
	Livestock station	Baidu Index (points)	+

In selecting the indicators, priority was given to data availability on the Baidu Index platform, relevance to rural information-seeking behavior, and the representativeness of each keyword within animal welfare dimensions. For the notifiable animal diseases dimension, “avian influenza” and “foot-and-mouth disease” were selected to reflect public risk perception of highly pathogenic animal diseases and concern for the basic welfare condition of “freedom from disease” (32). The common diseases dimension includes “pig diseases” and “chicken diseases,” which are closely tied to the everyday language of rural livestock keepers and signal continued public attention to frequent health issues in daily farming practices (48). For preventive measures, the keywords “animal quarantine” and “animal epidemic prevention” correspond to routine tasks performed by local veterinary workers and reflect public awareness of basic disease prevention and service efforts (49). Finally, in the service system dimension, “animal husbandry and veterinary” and “livestock station” were used to gauge how the public perceives the capacity, accessibility, and structure of grassroots veterinary services (50).

All eight keywords selected in this study are directly related to the “physical health” dimension of animal welfare, covering animal disease prevention and control, treatment of common diseases, vaccination behaviors, and veterinary service supply. These represent the fundamental baseline of animal welfare. The keywords “animal quarantine” and “animal epidemic prevention” also reflect standardized operational procedures, which may influence animals’ emotional states and are therefore indirectly linked to the “emotional and mental state” dimension. Meanwhile, “livestock station,” as a representative of local service platforms, supports the “expression of natural behaviors” through its role in feeding guidance and environmental management.

Overall, the PAAW subsystem establishes a measurable, structured, and rural-adaptive indicator framework, providing both data and conceptual foundations for the subsequent coupling coordination analysis with the VHR subsystem.

### 3.4 Subsystem evaluation

To ensure objectivity in weight assignment, this study applies the entropy weight method to calculate indicator weights for the VHR and PAAW subsystems, and to compute the scores for each province. The entropy method is a widely used way to assign weights based on data. It helps reduce personal bias and makes the results more reliable (51). However, the weights generated by this method may be sensitive to variations in data distribution and indicator selection, which could influence results. This limitation is further discussed in the limitations. The weights are calculated separately for each year to avoid interference from differences in data distribution across periods.

(1) Data standardization

To eliminate differences in scale and units among the indicators, the min–max normalization method is used to transform the data into a dimensionless form.

For a positive indicator:


(1) $$Y_{\mathrm{ij}}=\frac{X_{\mathrm{ij}}-\min(X_{\mathrm{ij}})}{\max(X_{\mathrm{ij}})-\min(X_{\mathrm{ij}})}$$

For a negative indicator:


(2) $$Y_{\mathrm{ij}}=\frac{\max(X_{\mathrm{ij}})-X_{\mathrm{ij}}}{\max(X_{\mathrm{ij}})-\min(X_{\mathrm{ij}})}\;$$

To avoid unreasonable outcomes where the minimum value results in a score of zero, all normalized values equal to 0 are replaced with a small constant (set at 0.01). This ensures that each indicator contributes at least minimally to the final score.

(2) To calculate the entropy:

(3) $$P_{\mathrm{it}}=\frac{Y_{\mathrm{ij}}}{\sum_{i=1}^{T}Y_{\mathrm{ij}}}$$

(4) $$e_{i}=-\frac{1}{\mathrm{lnT}}\sum_{i=1}^{T}P_{\mathrm{it}}\ln P_{\mathrm{it}}\;$$

(3) Apply weights to the indicators:

(5) $$w_{i}=\frac{1-e_{i}}{n-\sum_{i=1}^{n}e_{j}}$$

(4) The score of a subsystem for province *i* in year *t* is calculated as:

(6) $${\hat{Y}}_{t}=\sum_{i=1}^{n}w_{i}Y_{\mathrm{it}}$$

### 3.5 Panel vector autoregression model

This study assumes a causal relationship between VHR and PAAW, where both subsystems interact and evolve together within the animal health governance system. Coupling coordination requires dynamic linkages between systems rather than independent operation (52). Therefore, before assessing the level of coordination, it is necessary to determine whether interaction and causality exist between them.

To address this, the study applies a panel vector autoregression (PVAR) model. PVAR keeps the benefits of VAR in showing how things change over time and adds the strength of panel data to handle differences across regions and improve accuracy. This approach is well suited for exploring time-based interactions and causal links in multi-actor systems (53). By using the PVAR model, the study aims to uncover how VHR and PAAW influence each other over time, providing a solid foundation for the following coupling coordination analysis. The model is specified as follows:


(7) $$Y_{i,t}=A_{1}Y_{i,t-1}+A_{2}Y_{i,t-2}+\mu_{i}+\lambda_{t}+\varepsilon_{i,t}$$

Here, *Y_i,t_* = [*VHR_i,t_*, *PAAW_i,t_*]′ represents the panel vector of variables, *A_1_* and *A_2_* are the lag coefficient matrices to be estimated, *μ_i_* denotes region-specific fixed effects, *λ_t_* represents time fixed effects, and *ε_i,t_* is the disturbance term.

To ensure the validity of the PVAR model specification, unit root tests must be conducted before estimation to confirm that the variables are stationary and to avoid spurious regression. Levin–Lin–Chu (LLC) and Im–Pesaran–Shin (IPS) are two common methods used to test unit roots in panel data. Both can deal with time trends, unequal variances, and correlations across regions (54). LLC is suitable for homogeneous panels, while IPS allows for heterogeneous unit root processes, making it more flexible and widely applicable (55).

In addition to LLC and IPS, the augmented Dickey–Fuller (ADF) test was used as a robustness check. The ADF test evaluates the null hypothesis that a unit root exists in a time series, indicating non-stationarity. Rejecting the null supports the assumption of stationarity, which is a necessary precondition for valid dynamic modeling. Although commonly applied to time series, ADF helps validate the stationarity of variables and supports the reliability of panel-based results.

The optimal lag order is determined using several information criteria, including the Akaike Information Criterion (AIC), Bayesian Information Criterion (BIC), and Hannan–Quinn Information Criterion (HQIC). Based on the selected lag length, Granger causality tests and impulse response analysis are conducted. In the PVAR framework, the Granger test is used to identify the direction and strength of causality between VHR and PAAW (56). The impulse response function (IRF) is then applied to examine the time-path effects of external shocks on each variable, revealing the lagged response mechanism and duration of influence between the two systems (57).

### 3.6 Coupling coordination analysis

To quantify the interaction between VHR and PAAW, this study constructs a coupling coordination degree (CCD) model and a relative development degree (RDD) model to assess the coupling status of the two subsystems across provinces each year.

#### 3.6.1 Coupling coordination degree model

The coupling degree is widely used to reveal complex coupling mechanisms among systems (58). The formula is as follows:

(1) Coupling degree

(8) $$C=2\times {\left[\frac{U_{V}\times U_{A}}{\left(U_{V}+U_{A})(U_{V}+U_{A}\right)}\right]}^{1/2}$$

Here, C represents the coupling degree between the systems. *U_V_* and *U_A_* denote the comprehensive contribution values of VHR and PAAW to the combined system, respectively.

(2) Coupling coordination degree

The level of coupling alone does not reflect the development status of the two subsystems. A high coupling degree may still occur even when both VHR and PAAW are at low levels, which differs in meaning from high-level coordination. Therefore, a coupling coordination degree (CCD) model is introduced to evaluate how well the two subsystems develop in a balanced and coordinated manner. The CCD is calculated as follows:


(9) $$T={\mathrm{\alpha U}}_{V}+{\mathrm{\beta U}}_{A}$$

(10) $$D={\left(C\times T\right)}^{1/2}$$

Here, D represents the CCD, and T is the comprehensive coordination index, reflecting the joint contribution of VHR and PAAW to overall coordination. *α* and *β* are coefficients to be determined. Since VHR and PAAW are complementary and hold equal weight in their interaction, both *α* and *β* are set to 0.5.

(3) Classification of CCD

Following previous studies (59, 60), this study adopts a ten-level classification to interpret CCD values, ranging from extremely uncoordinated to perfectly coordinated. The classification is shown in Table 3. This approach allows for nuanced identification of coordination intensity across regions and time.

**Table 3:** Classification of the CCD between VHR and PAAW.

CCD (D)	0.0 ≤ D < 0.1	0.1 ≤ D < 0.2	0.2 ≤ D < 0.3	0.3 ≤ D < 0.4	0.4 ≤ D < 0.5
Coordination level	Extremely uncoordinated	Severely uncoordinated	Moderately uncoordinated	Slightly uncoordinated	Nearly uncoordinated
CCD (D)	0.5 ≤ D < 0.6	0.6 ≤ D < 0.7	0.7 ≤ D < 0.8	0.8 ≤ D < 0.9	0.9 ≤ D ≤ 1.0
Coordination level	Barely coordinated	Primarily coordinated	Moderately coordinated	Well coordinated	Perfectly coordinated

#### 3.6.2 Relative development degree model

To explore the internal constraints affecting the coordinated development of VHR and PAAW, this study introduces the relative development degree (RDD), denoted as R, which is defined as the ratio between VHR and PAAW.


(11) $$R=U_{V}÷U_{A}$$

Based on the RDD values of the two subsystems and drawing on previous studies (61, 62), the coupling development relationship between VHR and PAAW can be classified into three types: VHR lag (R < 0.9), VHR-PAAW synchronization (0.9 ≤ R ≤ 1.1), and PAAW lag (R > 1.1).

## 4 Results

### 4.1 Subsystem evaluation results

According to Equations 1–6, this study uses the entropy method to determine the weights of evaluation indicators and calculates the scores of VHR and PAAW for each province. The results are analyzed to reveal spatial patterns and regional disparities by comparing development levels across different areas.

#### 4.1.1 Patterns and evolution of VHR

**Figure 3:**
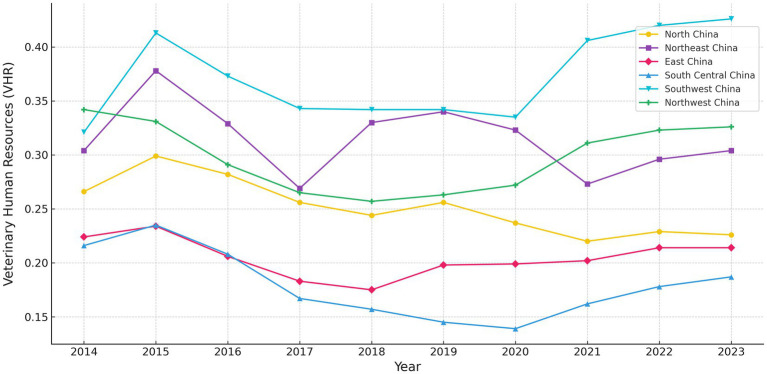
VHR level of China’s regions (2014–2023). For visualization purposes only, provinces are grouped into six traditional geographic regions. All statistical analyses are conducted at the provincial level.

As shown in Figure 3, there are clear regional differences in the development of VHR across provinces in China from 2014 to 2023. Some provinces—particularly in the southwest—demonstrate strong and consistent growth. For example, several provinces in this region reached their highest VHR levels in 2023, indicating robust workforce expansion in local veterinary institutions. In contrast, parts of northeastern and northern China experienced earlier peaks around 2015, followed by gradual declines or stagnation, suggesting weakening or plateauing capacity in workforce renewal. VHR levels in central and eastern provinces remain relatively low throughout the study period. Some of these areas reached their lowest points around 2020, likely due to shifting policy priorities or limited investment in grassroots animal health services. Meanwhile, certain western and northwestern provinces have shown a steady upward trajectory since 2018, reflecting progress in targeted rural veterinary workforce development.

**Figure 4:**
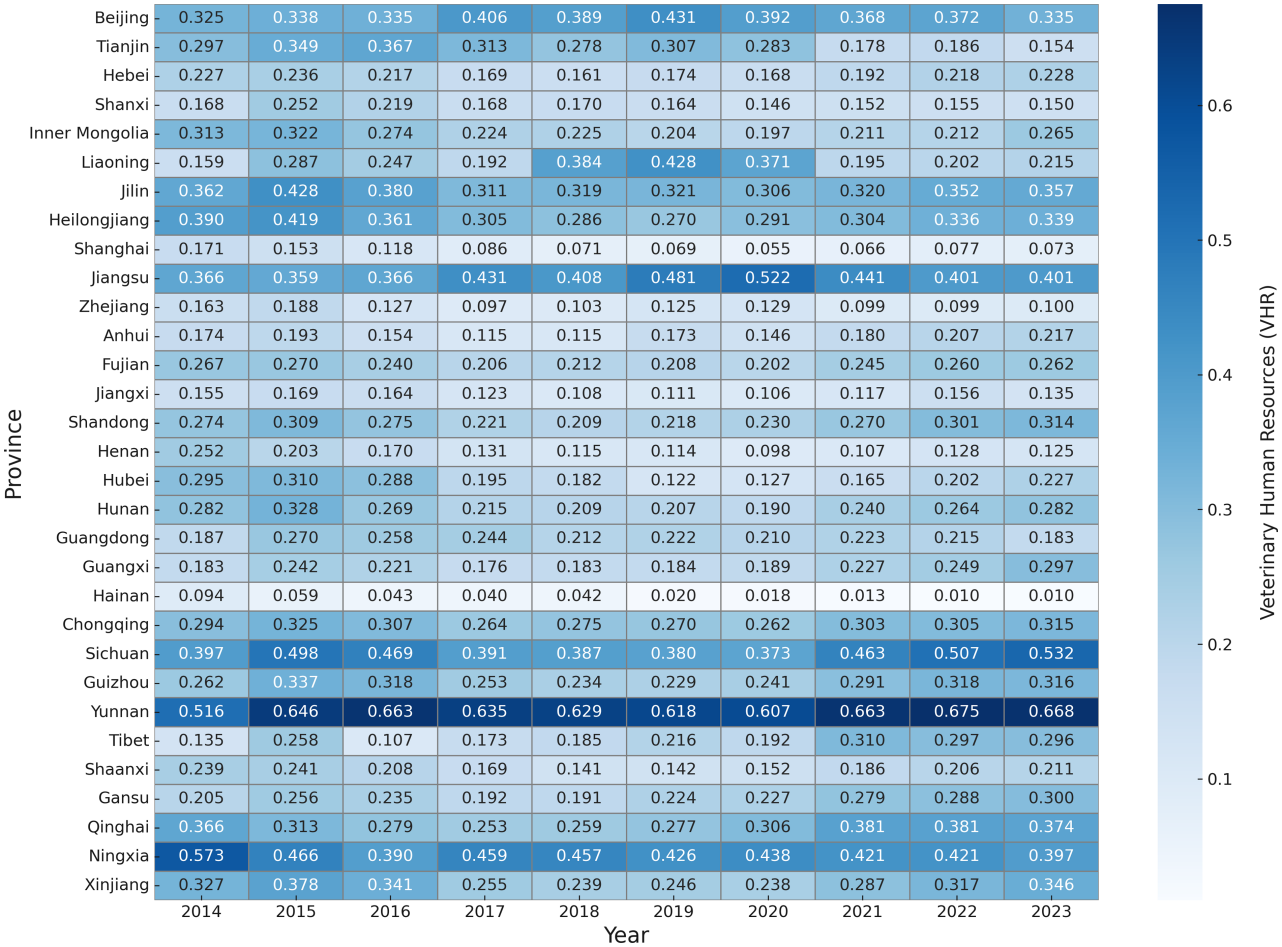
VHR level of China’s provinces (2014–2023). Each cell displays the VHR score for a given province and year, normalized to a 0–1 scale using the entropy weight method. Darker colors indicate higher VHR levels. Provinces are ordered vertically following standard national administrative divisions.

To provide a more detailed view, Figure 4 presents VHR scores at the provincial level. Provinces such as Yunnan and Sichuan consistently perform well, with Yunnan ranking first nationwide in several years and Sichuan showing steady growth since 2017. This reflects effective talent attraction mechanisms and sustained local investment. In contrast, economically developed eastern provinces such as Shanghai, Zhejiang, and Guangdong have relatively low VHR levels. This is not due to weak economic strength but rather to their industrial structure—animal husbandry accounts for a smaller share of their economy, which limits the actual demand for veterinary talent.

Meanwhile, regions like Ningxia, Guizhou, and Chongqing in central and western China have seen steady improvements, suggesting early progress in human resource development. However, provinces such as Hainan, Qinghai, Gansu, and Anhui continue to lag, with little sign of significant improvement. The northeastern provinces show considerable fluctuations and have not yet achieved stable development.

Overall, VHR development across the country follows a regional pattern: the Southwest leads, the East lags, the Northeast remains unstable, and the Central and Western regions are catching up. This reflects a structural imbalance in VHR development and highlights the urgent need for region-specific strategies to promote coordinated regional growth.

#### 4.1.2 Patterns and evolution of PAAW

**Figure 5:**
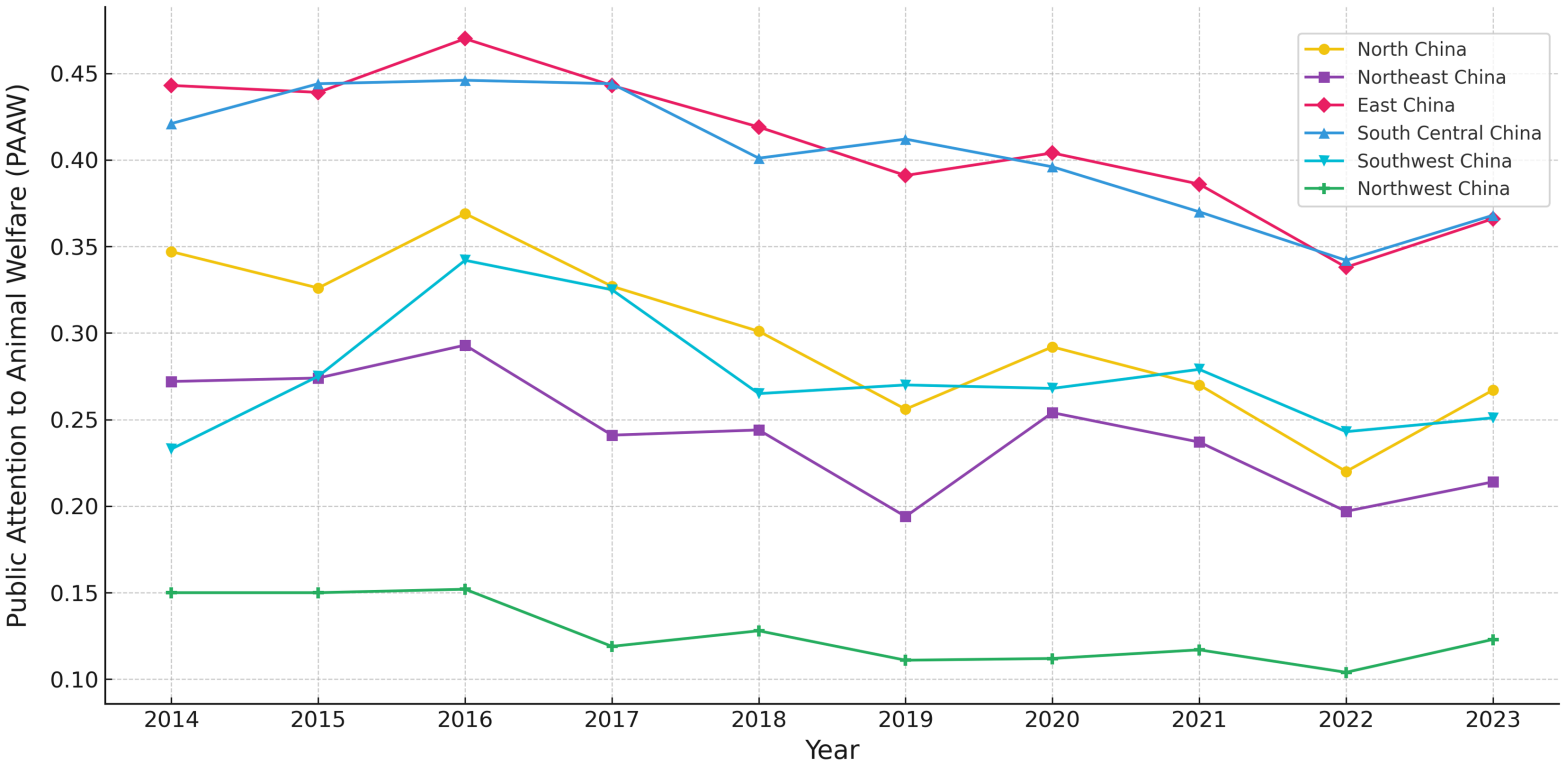
PAAW level of China’s regions (2014–2023). For visualization purposes only, provinces are grouped into six traditional geographic regions. All statistical analyses are conducted at the provincial level.

Based on Figure 5, the development of PAAW reveals significant disparities across provinces from 2014 to 2023. The East region consistently recorded the highest level of public attention, peaking in 2016, indicating a high level of public awareness and sensitivity to animal welfare issues. The Northeast region also showed relatively high levels, with a trend like the East region, though with slightly greater fluctuations. The North and South Central regions maintained moderate levels, with a slight decline in recent years, possibly due to changes in industrial structure and information dissemination frequency. The Southwest region showed lower levels of public attention with noticeable fluctuations, while the Northwest region remained at the lowest level over time, reflecting a serious lack of public awareness.

**Figure 6:**
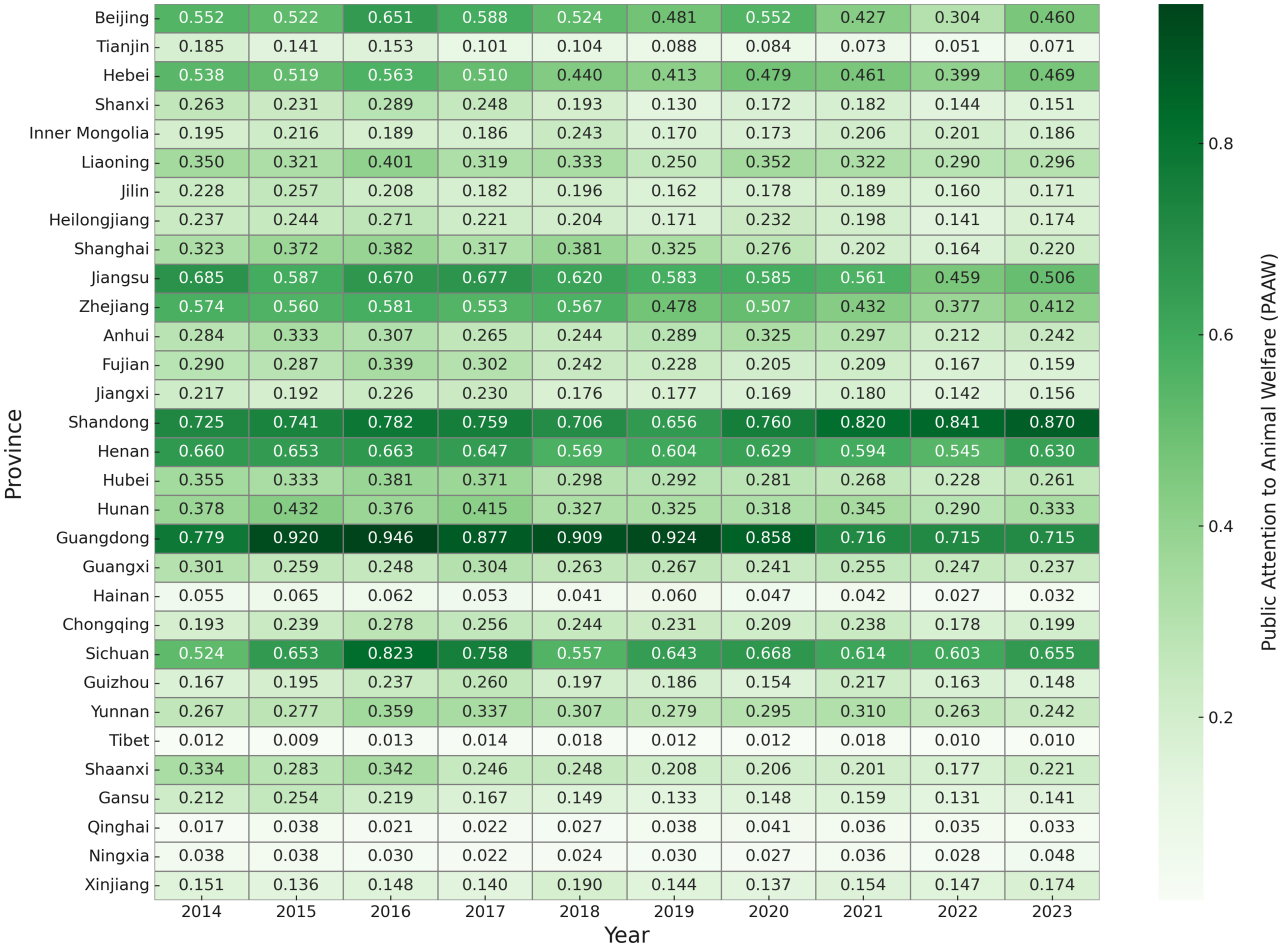
PAAW level of China’s provinces (2014–2023). Each cell displays the PAAW score for a given province and year, normalized to a 0–1 scale using the entropy weight method. Darker colors indicate higher PAAW levels. Provinces are ordered vertically following standard national administrative divisions.

To provide a more detailed spatial comparison, Figure 6 presents PAAW levels for each province and year. The overall distribution further confirms that eastern and southeastern provinces tend to have higher PAAW levels, while western and inland regions remain comparatively lower. Guangdong, Shandong, and Jiangsu consistently ranked among the top provinces, with Guangdong leading nationwide for several years. This reflects stronger public sensitivity to animal welfare issues and better access to information in economically developed areas. Attention levels were also relatively high in provinces such as Sichuan, Zhejiang, and Henan, suggesting that animal welfare awareness is gradually expanding in some central regions. In contrast, Tibet, Qinghai, Ningxia, and Hainan stayed at the low end throughout the period, showing continued gaps in information access, education, and local systems.

In general, the spatial differences in PAAW across regions come from many factors, including the level of economic development, access to information, public awareness, the quality of animal health service systems, and the distribution of the livestock industry. Future efforts should match local conditions and improve policies and outreach to help raise PAAW and support balanced development.

### 4.2 Causal relationship between VHR and PAAW

Based on Equation 7, this study uses PVAR model to check whether two subsystems are linked and if one causes changes in the other. Before analyzing how well VHR and PAAW work together, this study uses the PVAR model to test their causal relationship. The results are shown below.

#### 4.2.1 Stationarity test

This study first checks whether the VHR and PAAW scores are stable over time. ADF tests for each province are used to simulate the IPS method (see Table 4). The results show that 80.6% of the provinces pass the test for VHR at the 5% level, meaning the data are stable. For PAAW, 61.3% of the provinces pass the same test. Based on the rule that most provinces must pass for the data to count as stable, both VHR and PAAW are considered stable overall. So, they are suitable for the PVAR model in the next step.

**Table 4:** Stationarity analysis of VHR and PAAW.

Subsystem	Number of stationary provinces	Proportion	Stationarity status
VHR	25	80.6%	Yes
PAAW	19	61.3%	Yes

#### 4.2.2 Granger causality test

This study uses AIC, BIC, and HQIC to decide the number of lags. All three suggest using two lags. Based on this, a PVAR model with two lags is built. The Granger causality test and impulse response analysis are both carried out under this setting to check how stable and useful the model is. Table 5 shows the Granger test results for whether VHR and PAAW have a two-way causal link.

**Table 5:** Granger causality testing results of VHR and PAAW.

Original hypothesis	F-statistic	*p*-value	Conclusion
VHR is not the Granger cause of PAAW	≈ 8.05	≈ 0.02	Reject
PAAW is not the Granger cause of VHR	≈ 0.41	≈ 0.68	Do not reject

The results show that at the 5% level, VHR has a clear Granger causal effect on PAAW. This means that investing in VHR can help raise PAAW over time. On the other hand, the effect of PAAW on VHR is not clear. This suggests that public awareness alone is not strong enough to change the structure of veterinary services. These findings support the next step of impulse response analysis and provide a basis for building the coupling coordination model. They also show the direction in which VHR affects PAAW.

#### 4.2.3 Impulse response function analysis

**Figure 7:**
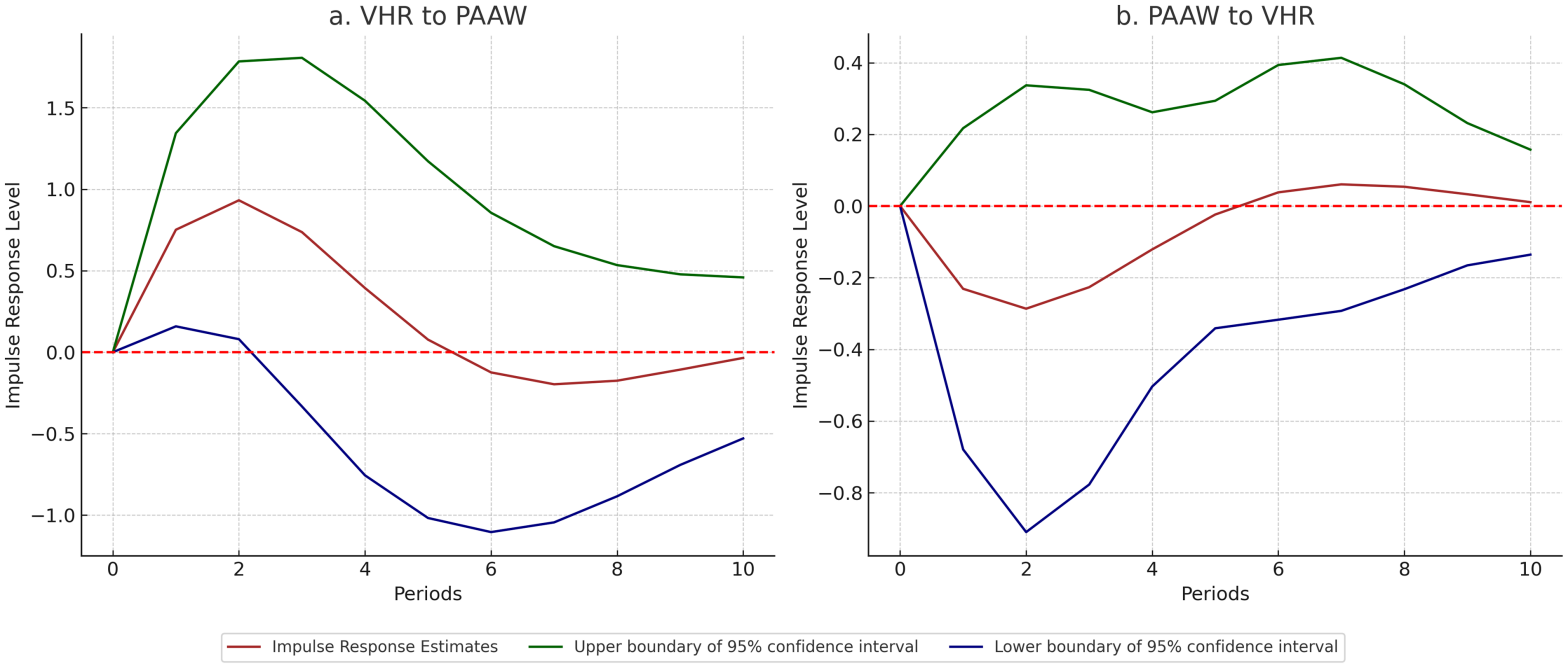
Impulse response of VHR and PAAW.

After confirming the Granger causality between VHR and PAAW, this study uses the impulse response function (irf) to explore how the two subsystems affect each other over time. Figure 7 shows the results of their two-way responses.

Findings indicate that a positive shock to VHR leads to a clear positive response in PAAW starting in the first period. The effect peaks in the second period, then gradually weakens and levels off after the fifth period. This suggests that VHR has a strong and sustained influence on PAAW in the medium term.

In contrast, the response of VHR to a shock from PAAW appears weak and volatile, with no consistent direction or pattern. While this supports the short-term unidirectional relationship identified by the Granger test, it does not necessarily rule out longer-term or indirect feedback effects. Institutional inertia or policy delays may obscure potential responses to public attention, which could become more apparent over longer time horizons. Further research could explore whether public attention exerts delayed or mediated influence on resource allocation through other channels.

In short, the IRF analysis confirms that VHR plays a leading and important role in raising PAAW. These results also provide evidence for the next step—analyzing the coordination between VHR and PAAW.

### 4.3 Coupling coordination results

#### 4.3.1 Coordination level analysis

**Figure 8:**
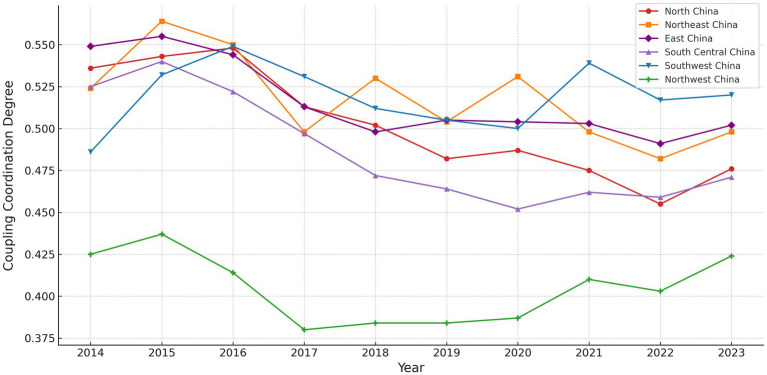
CCD level of China’s regions (2014–2023). For visualization purposes only, provinces are grouped into six traditional geographic regions. All statistical analyses are conducted at the provincial level.

Based on Equations 8–10, the CCD between VHR and PAAW is calculated, reflecting the extent to which public demand aligns with veterinary service capacity. As shown in Figure 8, for visualization purposes, CCD values are displayed by grouping provinces into six traditional geographic regions. While this provides a general sense of spatial distribution, all analytical interpretations are conducted at the provincial level.

From 2014 to 2023, CCD trends varied across provinces. The South Central region provinces remained in the barely coordinated state throughout the period, indicating limited but stable interaction between the two systems. The Northeast, North, and East regions reached relatively high levels around 2015 but then declined. The drop in the East was especially sharp, with some years falling to the lower end of the coordinated range. The Southwest region remained stable, with small fluctuations, and consistently stayed in the barely coordinated range. The Northwest region remained at a low level throughout the period. In some years, it fell into the nearly or slightly uncoordinated state, reflecting weak interaction between the two systems.

**Figure 9:**
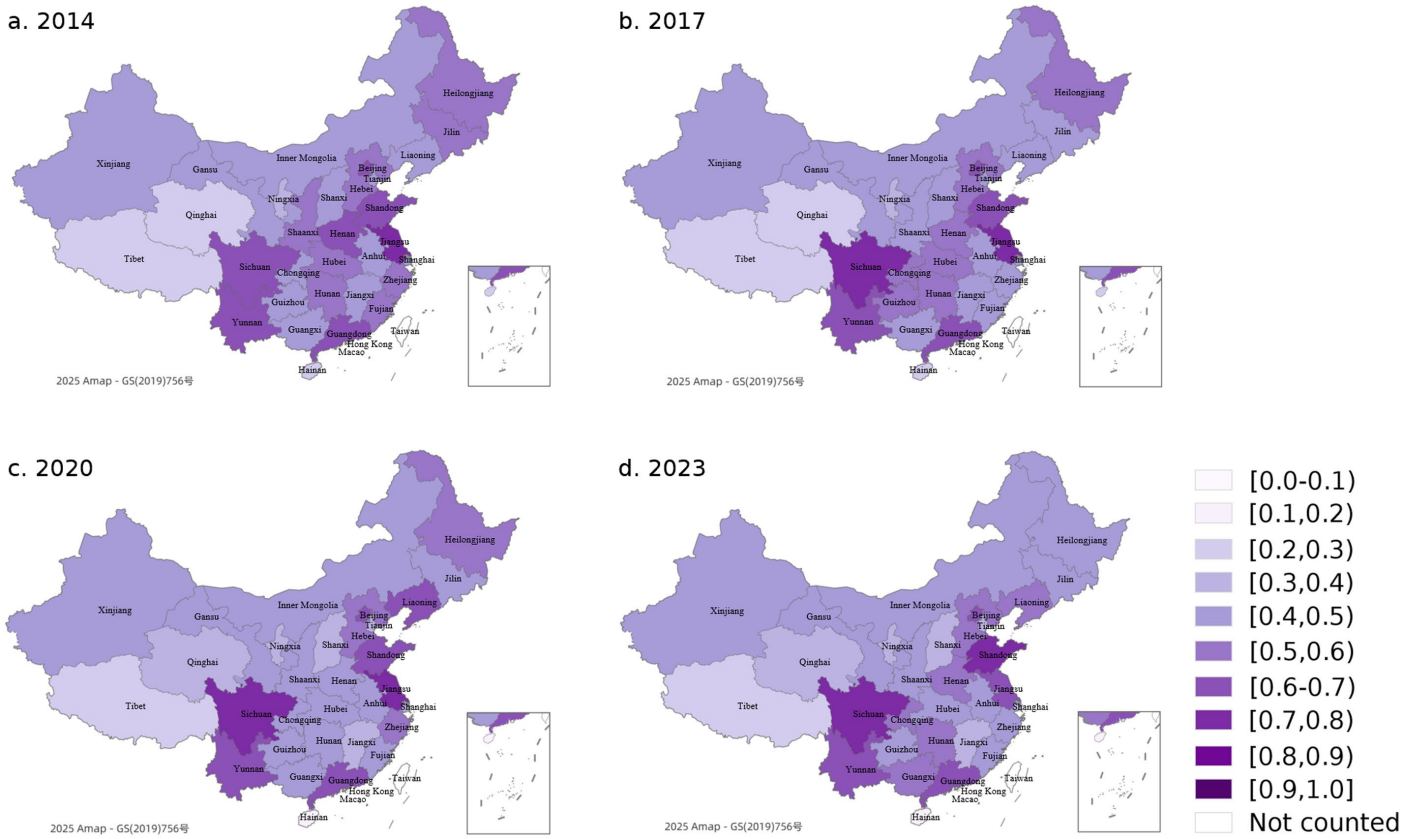
CCD level of China’s provinces. CCD between VHR and PAAW across China’s provinces in selected years (2014, 2017, 2020, and 2023). CCD values range from 0.0 to 1.0 and are classified into ten levels from “extremely uncoordinated” to “perfectly coordinated.” Darker colors indicate stronger coordination between the two subsystems.

A more detailed analysis at the provincial scale is presented in Figure 9, which shows CCD scores for selected years (2014, 2017, 2020, and 2023). The results indicate that most provinces remained in the “barely coordinated” to “moderately coordinated” range (between 0.5 and 0.8) throughout the observation period.

Provinces like Jiangsu, Shandong, Sichuan, and Yunnan maintained high CCD levels, mostly in the “Primarily” to “Moderately coordinated” range (between 0.6 and 0.8), with some years approaching “well coordinated” status. Beijing, Zhejiang, and Guangdong—despite being economically developed—showed relatively stable CCD. Though there were small fluctuations, they generally remained within the “barely” to “primarily coordinated” range (between 0.5 and 0.7).

In contrast, less developed western provinces such as Tibet, Qinghai, Ningxia, and Gansu remained mostly in the “Uncoordinated state” (below 0.5), indicating much weaker CCD performance between VHR and PAAW.

In summary, the CCD distribution across provinces reveals a clear spatial pattern. Provinces with stronger economic bases and more established veterinary systems tend to achieve better coordination between supply and demand. Conversely, provinces with limited investment in veterinary infrastructure or public awareness show persistently low coordination levels.

These findings underscore the need for province-specific strategies to strengthen the alignment between service capacity and public expectations. Improving CCD requires simultaneous attention to workforce development, digital outreach, and governance consistency—especially in provinces with historically weak performance. Tailored interventions in these areas will help promote a more balanced and integrated animal health governance system across China.

#### 4.3.2 Coordinated development patterns

**Figure 10:**
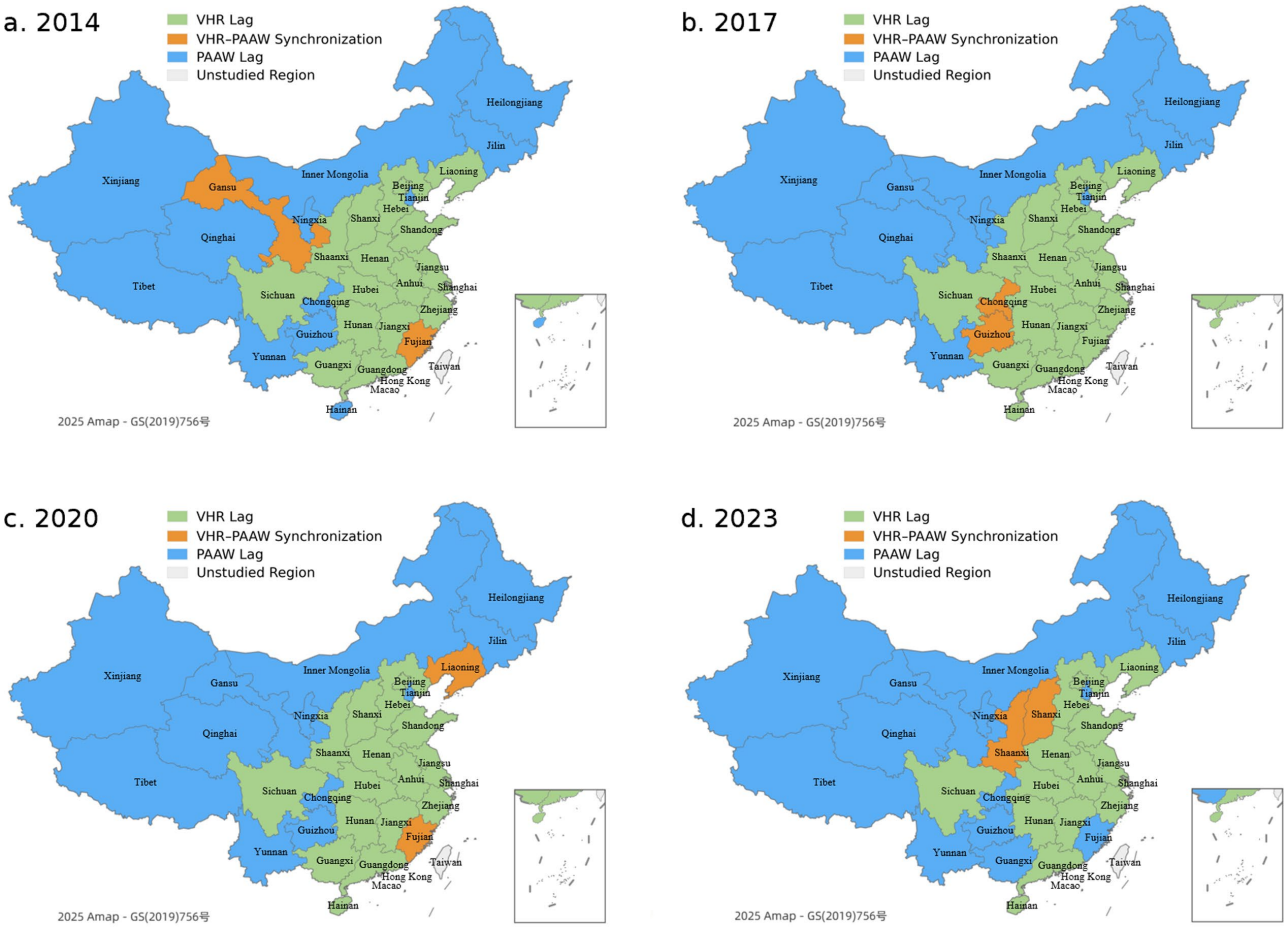
Coordinated development patterns of VHR and PAAW.

The relative development degree (RDD) is calculated using Equation 11. Based on the RDD classification, the coupling relationship between VHR and PAAW is divided into three types: VHR lag, PAAW lag, and VHR-PAAW synchronization (see Figure 10).

In terms of spatial and temporal patterns, the “VHR lag” type is the most common. It is widely found in South Central and East China, such as Henan, Hunan, Jiangxi, Anhui, and Fujian, as well as Sichuan in Southwest China. These provinces are characterized by intensive livestock production and rising PAAW. The persistent lag in VHR suggests that local veterinary services have not kept pace with growing public demand, especially in areas such as disease prevention, diagnosis, and health management.

The “PAAW lag” type mainly appears in the Northeast and Northwest regions, such as Heilongjiang, Inner Mongolia, and Gansu. Although these places have relatively strong veterinary capacity, low PAAW levels suggest challenges in information access, farming practices, or public awareness. The “VHR-PAAW synchronization” type appears only in a few provinces and specific years, such as Fujian, Liaoning, and Shaanxi. These cases often result from short-term factors like policy shifts, disease outbreaks, or media coverage. They tend to be temporary, which suggests that the coordination between supply and demand systems is not yet stable or sustained.

In summary, the overall relationship between VHR and PAAW shows a supply–demand mismatch. This reflects ongoing gaps between institutional support, resource allocation, service capacity, and public awareness. To achieve coordinated development, it is necessary to strengthen local veterinary service systems while also improving public understanding of animal welfare.

## 5 Discussion

### 5.1 Interpretation of key findings

This study assessed the development levels of VHR and PAAW across Chinese provinces from 2014 to 2023. It explored their causal relationship and coupling coordination patterns. The findings support some existing studies while also offering new insights.

#### 5.1.1 Spatial disparities in VHR and PAAW

In terms of VHR, the Southwest region—especially Yunnan and Sichuan—shows strong capacity in resource allocation and continued investment. These areas have made clear progress in attracting veterinary talent and building local service systems. In contrast, eastern developed provinces like Shanghai, Zhejiang, and Guangdong have relatively low VHR levels. This is mainly due to their industrial structure, which focuses on services and high-end manufacturing, with limited demand for veterinary services because of the smaller livestock sector. These findings reveal structural differences in China’s animal health system across regions (63).

Regarding PAAW, public attention remains high in coastal and northeastern regions. Provinces such as Guangdong, Shandong, and Jiangsu consistently rank at the top in Baidu search data. In contrast, PAAW levels are much lower in Central and West regions, especially in pastoral and less developed provinces. This shows clear regional disparities, shaped by differences in information access, education, institutional support, and economic development (29, 34, 64, 65).

#### 5.1.2 Unidirectional causal relationship between VHR and PAAW

The Granger causality test shows that VHR has a significant impact on changes in PAAW, while the reverse path from PAAW to VHR is not significant. This supports a supply-driven mechanism, in which stronger veterinary capacity contributes to increased public awareness of animal welfare (37). It also reflects the role of institutional capability in shaping public responsiveness (66).

The impulse response analysis reinforces this finding. When VHR receives a positive shock, PAAW responds quickly, reaches a peak, and then stabilizes. This shows that improvements in VHR can have a sustained effect on public engagement in the medium term. It supports earlier findings that information and resource supply can influence public digital behavior in its early stages (67).

However, the response from VHR to changes in PAAW is weak and unstable. This does not necessarily mean that public attention has no influence. Rather, such influence may take longer to manifest, possibly mediated through policy feedback mechanisms, budget cycles, or media-driven political agendas (68, 69). Therefore, while current evidence highlights the leading role of VHR, future research should consider longitudinal approaches or qualitative methods (e.g., policy tracing, stakeholder interviews) to examine whether public demand gradually feeds back into institutional adjustments over time.

#### 5.1.3 Regional disparities in VHR-PAAW coordination

In terms of regional patterns, provinces like Jiangsu, Shandong, and Yunnan show high levels of CCD. This suggests a good match between public resource allocation and public demand. In contrast, regions in the west, such as Tibet, Qinghai, and Ningxia, remain close to or within the range of imbalance. These areas show weak system interaction and poor resource integration. Such regional differences point to an uneven coupling mechanism between service capacity and public demand across provinces (33).

The analysis of RDD shows that most provinces fall into the “VHR lag” group, where PAAW grows faster than local veterinary service capacity. This reflects a common pattern in developing countries, where investment in human resources often falls behind rising public awareness of animal health (13). The “PAAW lag” type is mostly found in the Northeast and Northwest, where basic veterinary services are in place, but public attention remains low. This may result from limited access to information, livestock structure, and cultural acceptance, suggesting that digital access and social norms shape public awareness (70). The “VHR-PAAW synchronization” type appears only in a few provinces and usually in short periods. These cases are often driven by sudden policy actions or media events, leading to short-term changes in public attention (29), and a stable coordination mechanism has yet to form.

The regional disparities in CCD and RDD patterns can be explained by differences in institutional environments, governance models, and communication infrastructures. For instance, Yunnan exhibits relatively high CCD levels despite being a less economically developed province. This may be attributed to strong grassroots demand for animal health services in its livestock-intensive areas and provincial efforts to expand rural veterinary access (71). In contrast, Guangdong, despite its economic strength and developed infrastructure (72), shows only moderate CCD levels, suggesting that public attention and veterinary resource allocation may not be well aligned or equally prioritized across sectors.

In the Northeast and Northwest regions, where PAAW lag is common, the presence of veterinary systems has not translated into high public engagement. Factors such as limited information access, traditional farming practices, and cultural attitudes toward animal welfare may have contributed to weak public awareness (73). Meanwhile, provinces like Tibet and Qinghai remain in a persistently uncoordinated state, which may be associated with weaker institutional coordination and limited investment in rural service infrastructure. These findings suggest that beyond economic development, policy alignment and targeted public outreach play a critical role in shaping VHR-PAAW coordination.

Overall, VHR plays a leading role in driving changes in PAAW. However, structural imbalances and coordination gaps remain between regions. Unlike traditional approaches that treat supply and public behavior as separate issues, this study builds a joint framework linking VHR and PAAW. It reveals the dynamic interaction among institutions, resources, and public behavior. This offers a new way to understand structural mismatches in animal health systems and helps guide policy design.

### 5.2 Recommendations

The findings show that VHR plays a basic role in guiding PAAW. However, in most regions, there is still a gap between service supply and public awareness. To improve coordination, this study suggests action on talent supply, public engagement, and regional governance.

First, efforts should be made to strengthen local veterinary teams and improve both service access and professionalism. In regions like the West and Northeast, where VHR remains weak, more government support is needed. This includes restoring staffing plans for township veterinary stations, hiring more personnel, and making the jobs more attractive and stable. Training and continuing education should also be improved, especially to help young veterinarians work in rural areas. These steps can raise their ability in disease control, vaccination, and farming advice (74). Past studies have also stressed that talent stability and technical skills are key for strong public service systems (75). In addition, veterinary education should focus more on animal welfare and communication skills. This will help veterinarians better respond to public awareness (76).

Second, public awareness of animal welfare should be improved, and more ways for public engagement and feedback should be built. This study shows that public understanding of animal health and veterinary services varies by region. In places with less access to information, people usually pay less attention to animal welfare. It is suggested to use local platforms such as rural communities, schools, and cooperatives to promote basic knowledge about animal health and welfare. These efforts should match local language and communication habits to raise public awareness of risks and responsibilities (77). At the same time, tools such as satisfaction surveys, opinion collection, and complaint channels should be developed. Including public feedback in service management can make the system more responsive and open.

Third, region-based strategies are needed to match supply and demand. The study finds that regions differ greatly in their coordination level, causal patterns, and development stages. A single policy may not meet all needs. Local governments should design tailored measures based on local conditions (78). In VHR lag areas, service capacity should be improved quickly. In PAAW lag regions, value promotion and policy guidance are needed. Where VHR and PAAW grow together, but coordination is unstable, better service and management practices should be introduced. It is also helpful for local governments to use online behavior data to support policy making. This can help track social needs and react faster. Using data in this way has proven effective for making public services more sensitive and forward-looking (79).

### 5.3 Research significance

This study focuses on VHR and PAAW, assessing their development, relationship, and coordination. The findings respond to the practical question of how supply and demand connect in animal welfare, while contributing new theoretical and methodological insights to current research.

Theoretically, this study shifts from single-focus approaches to system-based analysis. Earlier studies often examined animals’ physical conditions, individual health, or ethical issues, but few explored the interaction between service supply and public awareness. By building a dual-subsystem framework, this research demonstrates that local veterinary resources play a crucial role in welfare governance. The integration of social behavior data with service systems provides fresh theoretical support and enhances understanding of how people, institutions, and public responses interact in animal health management.

Practically, the study identifies alignment between VHR and PAAW across Chinese provinces, revealing clear spatial differences. Many regions show insufficient VHR or low PAAW. By measuring coordination levels and development types, the study offers data-driven and regional strategies for improving veterinary services in China and provides insights for advancing One Health practices in other developing countries.

Methodologically, the study builds an integrated analysis framework combining indicator development, causal analysis, and coupling coordination assessment. Using diverse data sources and integrating the entropy method, PVAR model, and CCD model, it expands empirical tools for animal welfare research. This replicable approach can serve as a reference for studying supply–demand relationships in other public service areas, including rural healthcare and agricultural extension.

### 5.4 Limitations and future research

Although this study explores the dynamic link between VHR and PAAW through a structured framework, integrated data, and empirical modeling at the provincial level, a full understanding of their interaction and structural fit still requires further development in data, methods, and spatial coverage. Future research can move forward in several directions:

First, the data used still has some limitations. This study relies on publicly available sources such as statistical yearbooks and Baidu Index data. While these sources are representative and comparable, Baidu search data may disproportionately reflect the attention of younger, urban, and digitally connected users, while underrepresenting rural farmers, elderly individuals, and others with limited internet access. This may affect the comprehensiveness of the PAAW indicator, particularly in remote or less-connected regions. Future studies could consider triangulating Baidu Index data with other sources such as rural survey data, local media usage reports, or field interviews to better capture public awareness across diverse demographic and geographic groups (80). Additionally, the current keyword selection for PAAW relies mainly on formal or institutional terminology (e.g., “avian influenza”), which may not fully reflect how the public searches for related topics in everyday language. Colloquial terms such as “bird flu” or region-specific expressions could provide a more accurate picture of public interest. Future research should consider expanding the keyword list to include common vernacular and slang terms, thereby improving the inclusiveness and sensitivity of PAAW measurement.

Second, the study mainly uses quantitative methods, which limits the explanation of how the interaction works. While the models effectively capture statistical linkages and directionality, they do not fully account for the deeper institutional, cultural, or historical factors that shape these dynamics across regions. Additionally, the entropy weight method, although objective and widely used, is sensitive to the distribution and selection of indicators. Variability in input data can affect weight assignments and, consequently, influence subsystem scores and coupling results. These limitations highlight the need for mixed-methods approaches in future studies. Combining statistical models with qualitative tools—such as case comparisons, policy discourse analysis, or stakeholder interviews—can provide a more nuanced understanding of regional disparities and the underlying coordination mechanisms (81).

Third, the analysis is based on provincial-level data, which does not capture differences within each region. Using 31 provinces allows for national comparison, but it misses variation between cities, counties, and rural and urban areas. Future research could build datasets at the city or county level, where possible, to better reflect local patterns and give more detailed policy support to local governments.

## 6 Conclusion

This study integrates the VHR and PAAW subsystems to build a framework that links service supply and public awareness across 31 Chinese provinces from 2014 to 2023. It systematically evaluates their development levels, causal relationship, and coordination status. The results show clear regional imbalances: PAAW is stronger in the East region, VHR is weaker in Central and East regions, while the Southwest shows a relative lead. The PVAR model finds a one-way causal relationship from VHR to PAAW, suggesting that local service capacity plays a key role in shaping public awareness. CCD scores reveal that most regions remain at a primary or moderate level, with some areas showing structural mismatches between supply and demand.

On the theoretical side, this study brings veterinary services and public attention into one analysis framework, expanding animal welfare research from a single focus to a system-based approach. On the practical side, it measures how well local veterinary capacity aligns with public awareness, and identifies regional gaps to support targeted resource planning. On the methodological side, it proposes an interdisciplinary approach combining the entropy method, PVAR model, and CCD model, offering a replicable framework for empirical research in animal health governance. This framework may inform One Health governance in other developing countries.

However, the study has some limitations in terms of data sources, methods, and spatial scale. It does not fully uncover the micro-level mechanisms. Future work could bring in more data types and qualitative insights to deepen the understanding of how different actors interact across multiple levels, and to further improve the design and delivery of animal health services.

## Data Availability

Publicly available datasets were analyzed in this study. The data underlying this study are available at https://index.baidu.com (Baidu Index) and https://www.cnki.net/knavi/Yearbook.html (China Yearbooks Database).

## Footnotes

^1^ https://www.cnki.net/knavi/Yearbook.html

^2^ https://index.baidu.com
